# miR-7/EGFR/MEGF9 axis regulates cartilage degradation in osteoarthritis via PI3K/AKT/mTOR signaling pathway

**DOI:** 10.1080/21655979.2021.1988362

**Published:** 2021-10-18

**Authors:** Lifeng Jiang, Xindie Zhou, Kai Xu, Pengfei Hu, Jiapeng Bao, Jin Li, Junfeng Zhu, Lidong Wu

**Affiliations:** a Department of Orthopedics Surgery, The Second Affiliated Hospital, Zhejiang University School of Medicine, Hangzhou, China; b Department of Orthopedics, The Affiliated Changzhou No.2 People’s Hospital of Nanjing Medical University, Changzhou, China; c Department of Orthopedics Surgery, The Second Affiliated Hospital of Jiaxing University, Jiaxing, China; d Department of Orthopedics Surgery, Suichang Branch of the Second Affiliated Hospital, Zhejiang University School of Medicine (Suichang County People’s Hospital in Zhejiang Province), Suichang, LiShui, China

**Keywords:** Osteoarthritis, miR-7, EGFR, MEGF9, cartilage degradation

## Abstract

Osteoarthritis (OA) is a common degenerative disease in middle-aged and elderly people. Our previous study has proved that microRNA-7 (miR-7) exacerbated the OA process. This study was aimed to explore the downstream genes and mechanism regulated by miR-7 to affect OA. Multiple EGF-like-domains 9 (MEGF9) was the predicted target of miR-7 by databases. Luciferase report experiment results confirmed that MEGF9 could bind to miR-7. Among the 10 collected pairs of OA and healthy samples, the expression levels of miR-7 and MEGF9 were both up-regulated when compared with healthy subjects by qRT-PCR and immunohistochemistry (IHC). The increased MEGF9 levels were due to the interaction with epidermal growth factor receptor (EGFR) by co-immunoprecipitation. Evaluations found that upregulation of miR-7 or MEGF9 can increase the expression of EGFR, matrix metalloproteinase-13 (MMP-13) and a disintegrin like and metallopeptidase with thrombospondin type 1 motif 5 (ADAMTS-5), so as to aggravate cartilage degradation. In addition, this effect induced by miR-7/EGFR/MEGF9 axis was by activation of PI3K/AKT signaling. The IHC and western blot assay results on OA model mice also demonstrated that miR-7/EGFR/MEGF9 axis regulated cartilage degradation in vivo. In summary, miR-7/EGFR/MEGF9 axis may perform a crucial function in the regulation of OA, providing potential for OA treatment.

## Introduction

Osteoarthritis (OA) is a degenerative disease characterized by joint matrix destruction and articular cartilage cell reduction, leading to joint deformities and motor dysfunction [1,2]. In recent years, with the increase of aging population, the incidence of OA has also been increasing, and it has become a social problem that has attracted more and more attention [3]. The mechanism of OA is due to the reduction of proteoglycans, which leads to the synthesis and degradation of unbalanced cartilage matrix, thereby increasing the formation of osteophytes [4,5]. OA is also accompanied by the reduction of cartilage cells, leading to the destruction of articular cartilage, which is generally difficult to repair [6–9]. At present, there is no clear and effective cure for OA. Clinical treatment is mainly to control pain, improve joint function, and improve the quality of life of patients [10]. Conservative treatments for OA include pharmacologic therapy, injective therapy, supportive therapy, physical therapy, braces, or those and active exercise [11,12]. When conservative treatment fails, surgical treatments such as cartilage transplantation, joint cleaning, and joint replacement can be used [13]. Generally, pathogenesis of OA is a complex process of multi-factor and multi-step interaction, in which abnormal gene expression plays a significant role [14,15].

MicroRNAs (miRNAs) are a type of non-coding small RNAs that are ubiquitous in the genome of organisms and is only 20–24 nt in length [16]. Although miRNAs only account for about 1.0% of all RNAs, notably, they regulate 30%–50% of gene and participate in various cellular physiological processes such as cell proliferation, differentiation, and apoptosis [17,18]. Recently, the regulatory effects and mechanisms of miRNA-7 (miR-7) on a variety of cellular behaviors have become increasingly clear. Specifically, KEFAS et al. reported in 2008 that the gene sequence of miR-7 has at least 3 incomplete complementarities with the 3ʹ-UTR region of the epidermal growth factor receptor gene, and at least 2 in the 3ʹ-UTR region of AKT-2, and these regions are not completely complementary [19]. Liu et al. found that after gene transfection of miR-7 into the glioma U251 cell line, the PI3K/AKT pathway can be significantly inhibited, that resulting in decreased cell migration and increased apoptosis [20]. Not only that, with the in-depth studies, more and more evidences show that miRNA-7 (miR-7) is closely related to the onset and progression of various bone diseases such as osteoarthritis, osteoporosis, and bone tumors, but the exact mechanism of action has not been fully understood [21–23].

By analyzing the development mechanism of OA and the factors and pathways that may affect it, we found that the epithelial growth factor receptor (EGFR) is very important for tissue metabolism [24]. At the same time, studies have shown that the EGFR gene cartilage-specific knockout mice have cartilage surface defects and can spontaneously undergo cartilage degeneration. During the development of the growth plate, EGFR also plays an important regulatory role. And in the articular cartilage of OA patients and the articular cartilage of mouse OA models, it is found that there is a certain negative correlation between the activity of the EGFR pathway and the progression of OA [25,26]. In addition, we also found that PI3K/AKT/mTOR pathway can regulate the activation of many intracellular signal cascades and the function of various inflammatory mediators [27,28]. And studies have shown that in the onset of osteoarthritis, inhibiting the PI3K/AKT axis can prevent the inflammation of arthritic chondrocytes, the abnormal formation of bone degeneration, and the aging of cartilage cells [29–31]. On that basis, the expression level of miR-7 has a certain therapeutic effect in regulating cartilage degradation. Nevertheless, whether the mechanism of OA is related to the above-mentioned influencing factor, and how specific regulation mode works need to be elucidated urgently.

In this study, Starbase, TargetScan, and miRDB were utilized to predict the targets of miR-7. The intersection of the results provided us the target, multiple EGF-like-domains 9 (MEGF9), that was investigated for its effect on miR-7 related cartilage degradation in the course of OA.

## Methods and materials

### Collection of OA blood samples

After approved by the ethics committee, 10 OA samples and 10 healthy blood samples were obtained from the Second Affiliated Hospital of Zhejiang University School of Medicine. It is hereby explained that the OA samples mentioned above were all derived from the OA patients diagnosed in accordance with the guidelines for the diagnosis and treatment of osteoarthritis, combined with the clinical symptoms, signs, X-ray as well as laboratory tests, while the healthy blood samples were derived from healthy subjects. Afterward, related experiments were carried out with the knowledge and consent of the patients. Immediately after the blood sample was collected, it was centrifuged by Ficoll at 1500 rpm for 20 min, and the fraction containing monocytes was transferred to another tube, and Trizol was added to isolate and extract RNAs for reverse transcription.

### Chondrocytes culture and IL-1β stimulation

Human chondrocytes C28/I2 were cultured in DMEM medium containing 10% fetal bovine serum, 100 U/mL penicillin, and 100 U/mL streptomycin in a 37°C, 5% CO_2_ balanced humidity incubator. The chondrocytes in logarithmic growth phase were taken for experiment. The IL-1β (10 ng/mL) stimulation model was used to simulate the inflammatory environment of OA. The chondrocytes with miR-7 at a working concentration of 10 ng/mL were intervened, collected from different groups, and then used in this study.

### Quantitative real-time PCR

As mentioned in the literature, gene expression levels of miR-7, MEGF9, EGFR, MMP13, and ADAMTS-5 in chondrocytes from different groups were detected by quantitative real-time PCR (qRT-PCR). Specifically, the operation was followed by the steps of Trizol kit to extract total RNA from chondrocytes. Standard real-time fluorescence quantitative PCR was used for multiple detection. qRT-PCR was prepared according to the SYBR Green kit instructions, the expression level of target genes was calculated with 2^−ΔΔCT^ method, the RNA concentration was detected by Prime Script RT kit, and cDNA was synthesized by the reverse transcription kit. β-actin was used as an internal control. Each sample was tested three times to assure the reproducibility of the reaction.

### Cell transfection

The INTERFERin® transfection reagent was used for transfection followed by the instructions. The synthesized miRNA was transfected into chondrocytes via lipofectamine™ 2000, and the specific operation was carried out according to the instructions. Each sample was tested with five holes to assure the reproducibility. 48 h after transfection, the green fluorescence was observed under a fluorescence microscope to detect gene transfection efficiency.

### Western blotting

After collecting the chondrocytes 24 h after transfection, the total protein was routinely extracted, and protein concentration was determined by the BCA kit. Forty-microgram test protein was added to each well of the SDS-PAGE gel. Then, electrophoresis was performed at 110 V, 250 mA for approximately 2 h. After electrophoresis, the gels were sealed with 5% skimmed milk powder at 37°C for 1 h, and then the MEGF9, EGFR, MMP13, ADAMTS-5, p-PI3K, p-AKT, and β-actin primary antibodies were added, and incubated overnight at 4°C. After that, the membrane was washed with TBST for 3 × 10 min, the secondary antibodies were incubated at 37°C for 1 h, the membrane was washed with TBST for 3 × 30 min, and ECL was developed. Quantity one software was used to analyze the gray value of protein bands, and β-actin was used as an internal reference to calculate the relative expression level.

### Dual-Luciferase reporter assay

The bioinformatics online software starBase2.0 was used to predict target genes and analyze whether there are binding sites between MEGF9 and miR-7. The wild-type MEGF9 dual-luciferase reporter vector (WT MEGF9) and mutant MEGF9 dual-luciferase reporter vector (MUT MEGF9) were construct respectively, and then co-transfected with miR-7 mimic as well as the negative control into C28/I2 cells. After 48 h of culture, the luciferase activity was measured using a dual luciferase activity detection kit.

### Biotin-based RNA immunoprecipitation assay

As described as previous report [32,33], 3ʹ-biotinylated miR-7 mimics and 3ʹ-biotinylated mimics NC synthesized by GenePharma (Shanghai, China) and transfected into the HEK-293 T cells. Cells were lysed and the lysate was incubated with magnetic streptavidin beads. RNA was extracted from the beads and cDNA synthesis was performed. The binding between miR-7 and MEGF9 was detected by qPCR using the primers targeting the open reading frame (ORF) region or the 3ʹ-UTR of MEGF9.

### Co-immunoprecipitation

C28/I2 cells lysed by adding protease inhibitors and RNA lysis buffer that were included in the EZ-Magna RIP RNA-binding protein immunoprecipitation kit. The co-immunoprecipitation was performed according to the instructions. Subsequently, the anti-Argonaute2 (Ago2) antibody and normal mice IgG bound magnetic beads were incubated in RIP immunoprecipitation buffer, then, 100 μL of cell lysate was added. The samples were treated with proteinase K buffer while isolating the precipitated RNA. The concentration and quality of RNA was determined, and the purified RNA was analyzed by qRT-PCR method.

### Animal experiments

The IL-1β-induced destabilization of the medial meniscus model (DMM) was established to simulate OA according to the method reported in the literature [34]. A total of 30 four-week-old C57 male mice (18–22 g) were carefully resected without cartilage and ligament damage, and then randomly divided into six groups: the control group (no surgery; normal saline treatment; injection time as the OA) group; 10 knee joints from 5 mice, n = 10), OA group (surgery; normal saline treatment on the first day of each week from the 5th week to the 8th week after surgery, 10 knee joints from 5 mice, n = 10), OA miR-7-5p Up group (operation; 100 μL normal saline with 1 × 10^9^ PFU lentivirus vector of miR-7-5p Up; 10 knee joints from 5 mice, n = 10), OA miR-7-5p Up C Group (operation; 100 μL normal saline with 1 × 10^9^ PFU lentivirus vector of miR-7-5p Up Control, 10 knee joints from 5 mice, n = 10), OA miR-7-5p Down group (operation; 100 μL normal saline with 1 × 10^9^ PFU lentivirus vector of miR-7-5p Down;10 knee joints from 5 mice, n = 10), and OA miR-7-5p Down Group C (operation, 100 μL normal saline with 1 × 10^9^ PFU lentivirus vector of miR-7-5p Down Control, 10 knee joints from 5 mice, n = 10). Knee joint pathological specimens were taken at the 8^th^ weeks postoperatively, and the expression of MEGF9, EGFR, MMP-13, ADTMS-5 was detected by western blotting. Afterward, joint tissue sections were stained with Safranin O Fast Green, and the expression of MEGF9 was detected by immunohistochemistry. All animal-related experiments were conducted in accordance with the NIH guidelines (revised 1996), and the protocol was approved by the Ethics Committee of the Second Affiliated Hospital, School of Medicine, Zhejiang University, Hangzhou, China.

### Immunohistochemistry (IHC)

Specifically, after approved by the ethics committee, mice OA subjects were injected with miR-7 up, miR-7 up control, miR-7 down, miR-7 down control, and the normal group and the OA group in the joint cavity, respectively. The cartilage tissues from different groups were fixed with 4% paraformaldehyde for 48 h, decalcified with 10% hydrochloric acid, dehydrated with alcohol, embedded in conventional paraffin, and then cut into 4 μm sections (3 sections for each subject), stained with hematoxylin and eosin. Subsequently, the expression level of MEGF9 in the cartilage tissues of the knee joints was observed under a microscope. Random regions in the sections were selected to analyze.

### Statistics

Data were performed as mean ± SD with at least three replicates in each experiment. Using the Student's t-test method to analyze the two independent groups. The one-way ANOVA and Tukey tests were applied to analyze the differences among three groups or more. Differences were considered to be significant at *P* < 0.05.

## Results

### MEGF9 was the target of miR-7 and is positively related to the expression of miR-7

**Figure 1.**
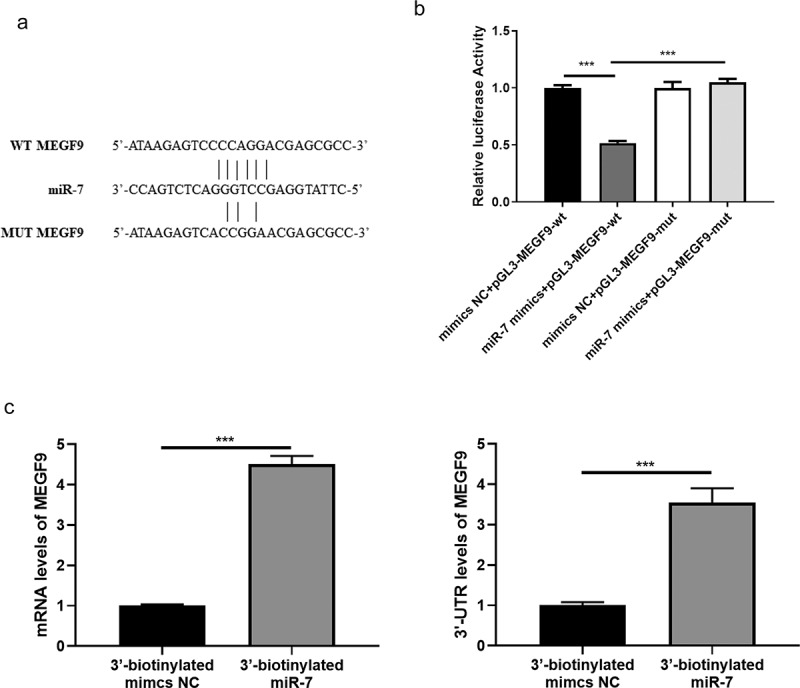
MEGF9 is a target of miR-7. (a) The sequence of MEGF9 contains a nucleotide sequence complementary to miR-7. (b) Dual‐luciferase reporter assay and (c) Biotin-based RNA immunoprecipitation assay for binding interaction between miR-7 and MEGF9. ***P < 0.001

Based on the target intersection obtained from Starbase, TargetScan, and miRDB, MEGF9 was considered as the most promising target of miR-7. The binding site between MEGF9 and miR-7 is displayed in Figure 1(a). And the results of the dual-luciferase report experiment showed that the luciferase activity of the wild-type MEGF9 could be significantly reduced compared to the mutant MEGF9 (Figure 1(b)). To provide more evidence for the binding between miR-7 and MEGF9, biotin-based RNA immunoprecipitation assay was performed. As shown in Figure 1(c), the significantly higher levels of ORF region and 3ʹ-UTR of MEGF9 were exhibited in 3ʹ-biotinylated miR-7 mimics compared to 3ʹ-biotinylated mimics NC group. These findings indicated the binding between miR-7 and the 3ʹ-UTR of MEGF9.

**Figure 2.**
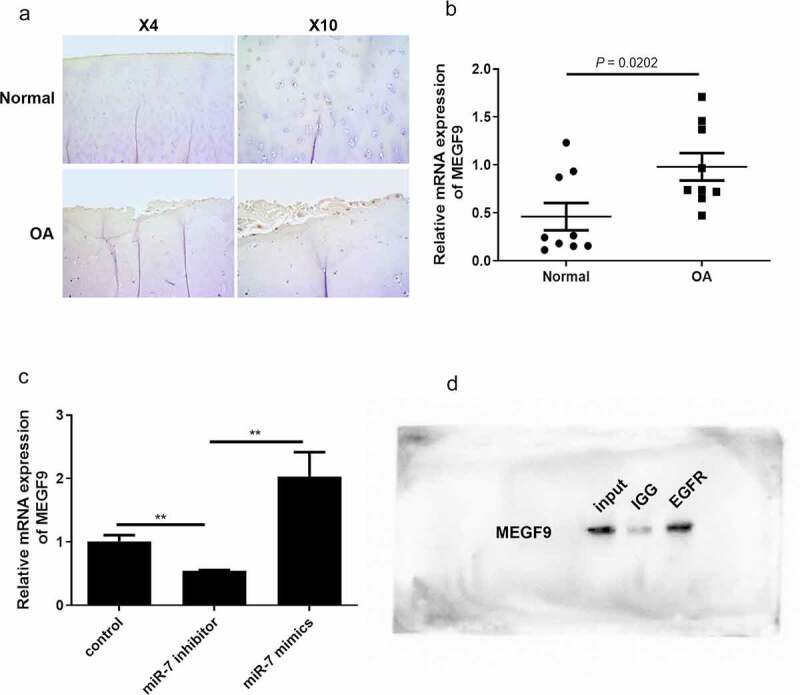
MEGF9 is higher expressed in OA tissues and interacts with EGFR

In order to analyze the correlation between miR-7 and MEGF9 in clinical OA samples and OA cells, the level of MEGF9 was detected by immunohistochemistry and qRT-PCR. The IHC results in Figure 2(a) demonstrate that MEGF9 was significantly higher expressed in OA samples compared to normal ones. The qRT-PCR results shown in Figure 2(b) also indicate that compared with 10 healthy subjects, the expression of MEGF9 was significantly up-regulated in 10 OA patients (*P* = 0.0202). Besides, our previous study has demonstrated that miR-7 level was significantly higher in OA patients than that in healthy subjects [35]. In the IL-1β-induced chondrocytes C28/I2 cells (OA cells), miR-7 mimics could significantly elevate MEGF9 level, while miR-7 inhibitor exhibited the opposite effect (Figure 2(c)). MEGF9 with multiple EGF-like repeats, consists of an N-terminal region with several potential O-glycosylation sites followed by five EGF-like domains, suggesting that there was interaction between MEGF9 and EGFR [36]. The CO-IP assay result shown in Figure 2(d) was to confirm that there is an interaction between EGFR and MEGF9. Besides, EGFR has been reported with significant correlation of OA. All of these findings revealed that MEGF9 interacted with EGFR, and was up-regulated in OA, that positively related to miR-7 level.

(a) Immunohistochemical results and (b) qRT-PCR results of MEGF9 in OA and healthy tissues. (c) After miR-7 expression was inhibited, the expression level of MEGF9 would be significantly reduced. (d) Co-immunoprecipitation results of MEGF9 and EGFR. **P < 0.01.

### Effect of miR-7 on the expression of MEGF9, EGFR, MMP-13, and ADAMTS-5 in articular chondrocytes induced by IL-1β

**Figure 3.**
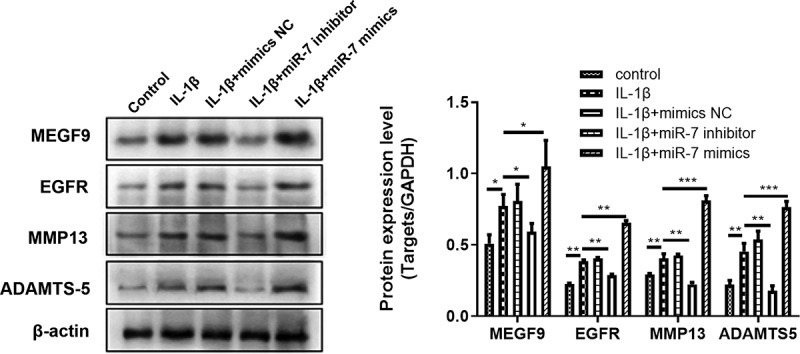
miR-7 regulates MEGF9 to affect cartilage degradation related proteins. The western blot and quantitative analysis of MEGF9, EGFR, MMP13 and ADAMTS-5 expression regulated by miR-7 in OA cells with different treatments. *P < 0.05, **P < 0.01, ***P < 0.001

The effect of miR-7 on MEGF9, EGFR, MMP13, and ADAMTS-5 expression in 10 ng/mL IL-1β-induced chondrocytes was evaluated by qRT-PCR. The results presented in Figure 3 reveal that when compared with the normal control group, the expression of miR-7, MEGF9, EGFR, MMP13, and ADAMTS-5 in IL-1β group was significantly increased, respectively. In addition, when compared with the IL-1β group, the levels of these targets were significantly reduced by adding miR-7-siRNA, while miR-7-mimics contributed the opposite effect, that significantly elevated these levels compared to IL-1β group. The same trend was displayed in western blot assay as shown in Figure 3.

### Effect of MEGF9 on the expression of EGFR, MMP-13, ADTMS-5, and PI3K/AKT signaling pathway

**Figure 4.**
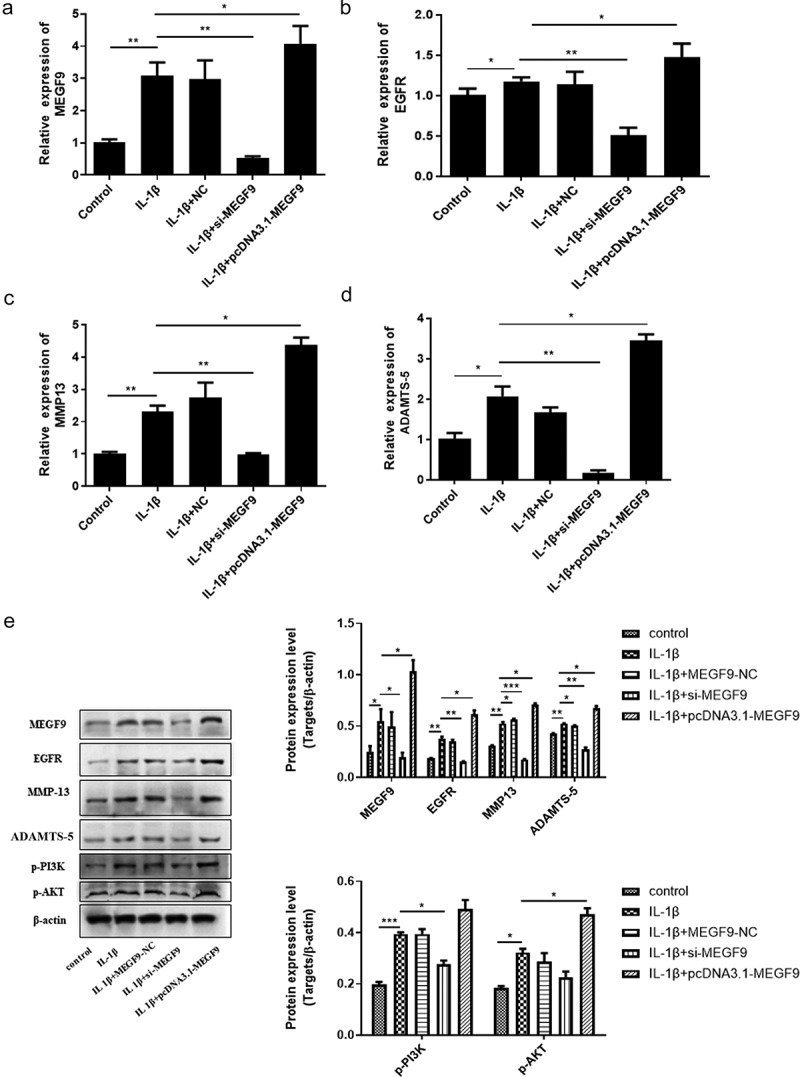
Overexpression of MEGF9 promotes cartilage degradation and activates PI3K/AKT signaling pathway. The mRNA levels of MEGF9 (a), EGFR (b), MMP13 (c), and ADAMTS-5 (d); and the western blot and quantitative analysis (e) of MEGF9, EGFR, MMP13, ADAMTS-5, p-PI3K and p-AKT in OA cells with different treatments. *P < 0.05, **P < 0.01, ***P < 0.001

To figure out whether MEGF9 play a role in miR-7-mediated cartilage degradation, chondrocytes were divided into the following groups: control, IL-1β, IL-1β +NC, IL-1β+ si-MEGF9, IL-1β+pcDNA3.1-MEGF9, and the expression of MEGF9, EGFR, MMP-13, and ADTMS-5 was evaluated by qRT-PCR and western blot. Results as shown in Figure 4(a-e) exhibited that in mRNA and protein level, when compared with the control group, all the measured indicators (MEGF9, EGFR, ADTMS-5, and MMP13) of the IL-1β group were significantly increased. When compared with IL-1β, the expression of each measured indicators in the IL-1β+si-MEGF9 group was significantly reduced, while the expression of each one in the IL-1β+pcDNA3.1-MEGF9 group increased. It also proved that PI3K/AKT signaling pathway was significantly activated in IL-1β group compared to control group, si-MEGF9 weakened this activation, and pcDNA3.1-MEGF9 strengthened this signaling.

**Figure 5.**
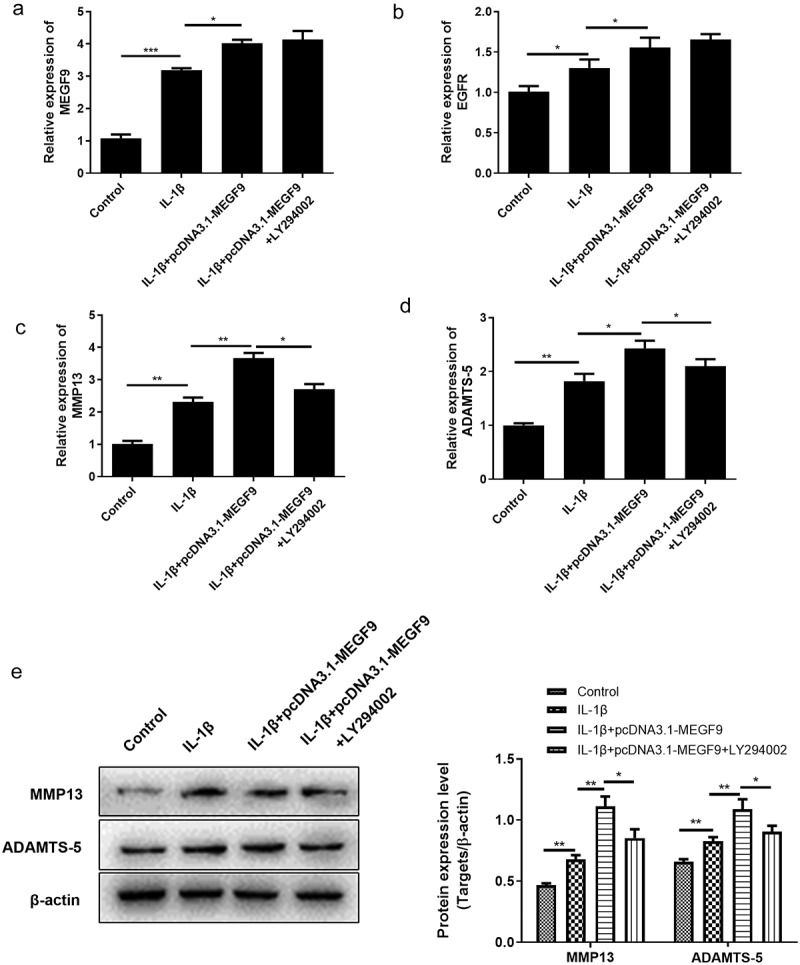
LY294002 rescues the effect of MEGF9 on cartilage degradation. The mRNA levels of MEGF9 (a), EGFR (b), MMP13 (c), and ADAMTS-5 (d); and the western blot and quantitative analysis (e) of MMP13 and ADAMTS-5 in OA cells with different treatments. *P < 0.05, **P < 0.01, ***P < 0.001

Subsequently, we explored whether the mechanism of MEGF9’s action is dependent on PI3K/Akt pathway regulation. The PI3K inhibitor, LY294002, was added in the cells to prove the rescue effect. As results shown in Figure 5(a-d) revealed that when chondrocytes were co-incubated with LY294002, the expression levels of MEFG9 and EGFR had no change compared to IL-1β+pcDNA3.1-MEGF9 group, suggesting that PI3K/Akt signaling could not alter the expression of MEGF9 and EGFR. However, the high levels of downstream genes related to cartilage degradation, MMP13 and ADTMS-5 induced by pcDNA3.1-MEGF9 were weakened by adding LY294002, indicating that the effect of MEGF9 on cartilage degradation genes was dependent on PI3K/Akt signaling. The similar trend was also displayed in protein level as shown in Figure 5(e).

### miR-7 mediated MEFG9 to regulate the expression of cartilage degradation-related proteins in OA mice

**Figure 6.**
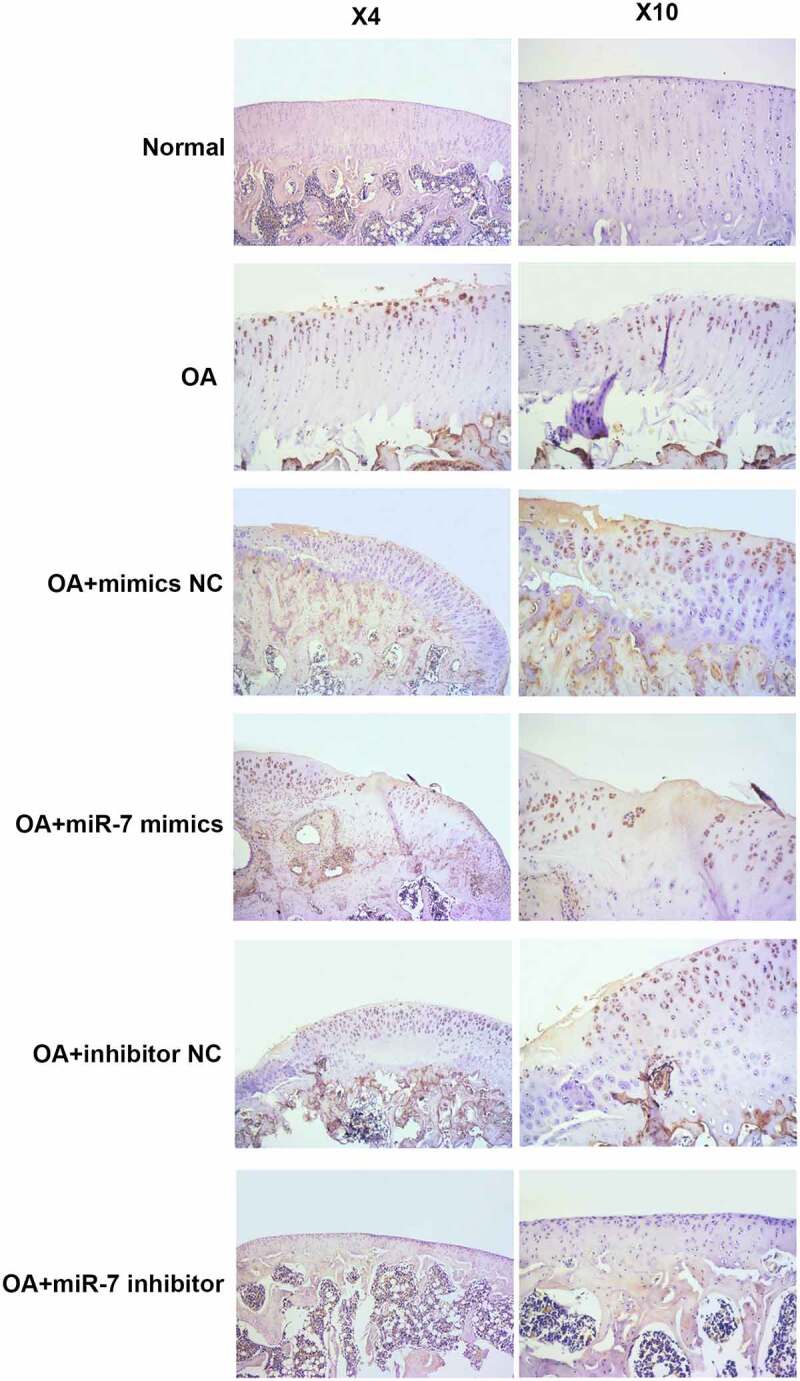
MEGF9 was highly expressed in OA mice with miR-7 mimics treatment. The immunohistochemical analysis of MEGF9 in OA mice

**Figure 7.**
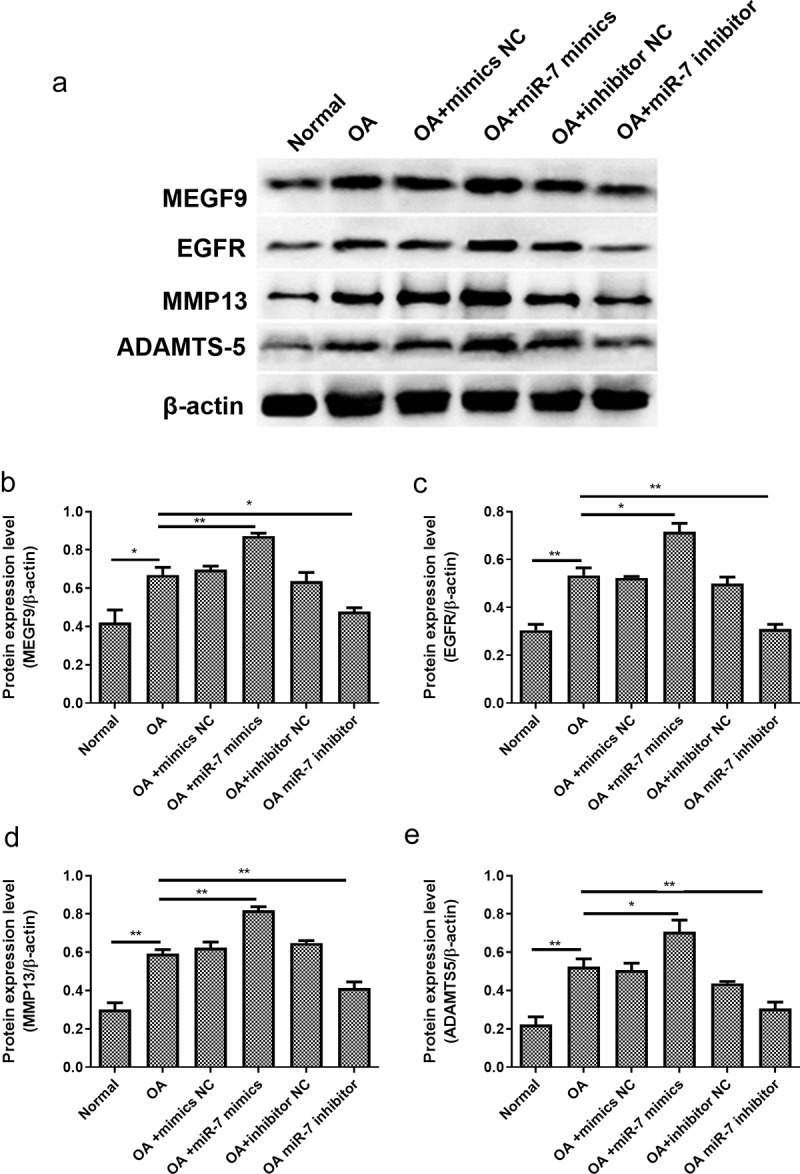
miR-7 mediated MEFG9 to regulate the expression of cartilage degradation-related proteins in OA mice. (a) The western blot results of MEGF9, EGFR, MMP13, and ADAMTS-5; and quantitative analysis of MEGF9 (b), EGFR (c), MMP13 (d) and ADAMTS-5 (e) in OA mice with different treatments. *P < 0.05, **P < 0.01, ***P < 0.001

In order to verify these findings in vivo, OA mice models were utilized. The OA mice were injected with different plasmids into the joint cavity. The cartilages were collected for histological evaluation. The immunohistochemical analysis of MEGF9 displayed in Figure 6 shows that, when compared with the OA group, the relative optical density of MEGF9 in the OA+miR-7 inhibitor group was significantly lower, while that of the OA+miR-7 mimics group was significantly higher than OA group as well as NC group. Western blotting assays were carried out, and the results are shown in Figure 7. When compared with the normal group, the expressions of MEGF9, EGFR, MMP-13, and ADAMTS-5 in all OA groups increased significantly. When compared with the OA group, the targeted proteins’ expression of the OA+miR-7 mimics group and OA+miR-7 inhibitor group were significantly increased and decreased, respectively. Based on the results, it was concluded that in the OA mice, the miR-7 would mediate increased MEGF9 level, thereby upregulating cartilage degradation-related protein levels and aggravating cartilage degradation in vivo.

## Discussion

Osteoarthritis (OA) is the most common chronic degenerative disease of joints [37]. Although effective treatments to delay the progression of osteoarthritis are still lacking, studies aiming to identify novel therapeutic targets were conducted to elucidate the specific molecular basis of OA progression. Chondrocyte hypertrophy is believed in playing a very crucial role in the initial and developmental stages of articular cartilage degradation [38]. The hypertrophy of chondrocytes is accompanied by the expression of some decomposing factors in articular cartilage, such as MMP-13, ADAMTS-5, etc., which then destroy the homeostasis of adult articular cartilage. Recently, more and more studies have shown that miRNAs play an important role in the occurrence and development of OA. For example, miR-103 promotes the development of OA by targeting Sox6, and miR-29 targets VEGF to reduce OA symptoms [39]; miR-138-5p/SIRT1 regulates chondrocytes ATDC5 and CHON-001 from IL-1β-induced inflammation [40].

Particularly, our previous findings demonstrated that the ciRS-7/miR-7 axis can possibly serve as a regulator in mediating proliferation, apoptosis, and inflammation in chondrocytes in the process of OA development [35]. In this further study, the potential therapeutic target genes of miR-7 for OA were explored. Based on the prediction results, MEGF9 was considered the promising one. Mutation of MEGF9 has been related to several disorders such as Marfan syndrome [41] and leukoencephalopathy [42], and decreased expression of MEGF9 in mesenchymal tumors may be associated to tumor local invasion [43]. LLeonart et al. reported that knockdown of MEGF9 may provide a novel approach for breast cancer treatment [44]. The exact role of MEGF9 mediated by miR-7 in OA is still unclear, so as to encourage us to do further explorations. In this study, it was found that miR-7 is highly expressed in OA tissues and cells, MEGF9 is also highly expressed, that was positively related to miR-7 level. The COIP result demonstrated that the interaction between EGFR and MEGF9, that contributed to the higher level in OA tissues, suggesting that miR-7/EGFR/MEGF9 axis might be functionally involved in the occurrence and development of OA.

As 10 ng/mL IL-1β can successfully induce OA environment in vitro, the chondrocyte proliferation would be inhibited, and the release of inflammatory cytokines and apoptosis would be promoted [45]. Destruction of extracellular matrix (ECM) homeostasis is a key event in the pathogenesis of OA. Cartilage degradation-related MMP-13 and ADAMTS5, those can degrade various ECM components, are two key factors in ECM homeostasis [46,47]. In the OA cells, it was proved that miR-7 was in charge of MEGF9 expression, and miR-7/EGFR/MEGF9 axis could regulate the levels of MMP-13 and ADAMTS5, contributing to the OA progress. Higher miR-7, accompanied with higher MEGF9, resulted in higher levels of MMP-13 and ADAMTS5, that aggravating OA.

Moreover, studies have found that the PI3K/AKT/mTOR pathway plays a key role in delaying inflammation, and also regulates the chondrocyte apoptosis, metabolism, gene expression, etc. [48,49], that is essential for normal metabolism of joint tissues, but is also involved in development of OA [48]. Our findings revealed that miR-7/EGFR/MEGF9 axis affected the activation of PI3K/AKT signaling pathway. The increased expression of MEGF9 could intensify the phosphorylation levels of PI3K and AKT. When OA cells were co-cultured with PI3K inhibitor LY294002, the high levels of MMP-13 and ADAMTS5 induced by MEGF9 could be weakened, indicating that the effect of MEFG9 on cartilage degradation-related proteins was dependent on PI3K/AKT signaling. To further demonstrate the effect of miR-7/EGFR/MEGF9 axis, the OA model mice were utilized. The results provided the evidence that the downstream target of miR-7, MEGF9, was upregulated by miR-7 mimics, and increased the levels of MMP-13 and ADAMTS5, that exacerbating cartilage degradation in vivo.

## Conclusions

In summary, it was discovered by this study that miR-7/EGFR/MEGF9 axis played a role in cartilage degradation of OA, that providing a novel insight for OA treatment. In the future, the specific application of miR-7/EGFR/ MEGF9 axis could be utilized in OA.

## Limitations

Although the effect of miR-7/EGFR/MEGF9 axis has been explored in cartilage degradation of OA, the underlying mechanisms by which MEGF9 affect PI3K/AKT signaling and the other in-depth mechanism of this axis in OA should be investigated in vitro and in vivo, that contributed more evidence for the axis in OA treatment.

## Data Availability

The data that support the findings of this research are available from the corresponding author upon reasonable request.

## Highlights

1. Multiple EGF-like-domains 9 (MEGF9) was the target of miR-7 in OA.
2. Overexpression of MEGF9 aggravates cartilage degradation by activating PI3K/AKT signaling.
3. miR-7/EGFR/MEGF9 axis provided potential for OA treatment.
